# Bariatric-induced microbiome changes alter MASLD development in association with changes in the innate immune system

**DOI:** 10.3389/fmicb.2024.1407555

**Published:** 2024-08-09

**Authors:** Simer Shera, William Katzka, Julianne C. Yang, Candace Chang, Nerea Arias-Jayo, Venu Lagishetty, Anna Balioukova, Yijun Chen, Erik Dutson, Zhaoping Li, Emeran A. Mayer, Joseph R. Pisegna, Claudia Sanmiguel, Shrey Pawar, David Zhang, Madelaine Leitman, Laura Hernandez, Jonathan P. Jacobs, Tien S. Dong

**Affiliations:** ^1^The Vatche and Tamar Manoukian Division of Digestive Diseases, Department of Medicine, David Geffen School of Medicine at UCLA, Los Angeles, CA, United States; ^2^UCLA Microbiome Center, David Geffen School of Medicine at UCLA, Los Angeles, CA, United States; ^3^Department of Surgery, UCLA Center for Obesity and METabolic Health (COMET), Los Angeles, CA, United States; ^4^David Geffen School of Medicine at UCLA, Los Angeles, CA, United States; ^5^Division of Gastroenterology, Hepatology and Parenteral Nutrition, VA Greater Los Angeles Healthcare System, Los Angeles, CA, United States; ^6^UCLA Center for Human Nutrition, University of California, Los Angeles, Los Angeles, CA, United States; ^7^G. Oppenheimer Center for Neurobiology of Stress and Resilience, University of California, Los Angeles, Los Angeles, CA, United States

**Keywords:** metastatic dysfunction-associated steatotic liver disease, obesity, host innate immune system, microbiome, fecal transplant

## Abstract

**Introduction:**

Metabolic dysfunction-associated steatotic liver disease (MASLD) affects nearly 25% of the population and is the leading cause for liver-related mortality. Bariatric surgery is a well-known treatment for MASLD and obesity. Understanding the fundamental mechanisms by which bariatric surgery can alter MASLD can lead to new avenues of therapy and research. Previous studies have identified the microbiome’s role in bariatric surgery and in inflammatory immune cell populations. The host innate immune system modulates hepatic inflammation and fibrosis, and thus the progression of MASLD. The precise role of immune cell types in the pathogenesis of MASLD remains an active area of investigation. The aim of this study was to understand the interplay between microbiota composition post-bariatric surgery and the immune system in MASLD.

**Methods:**

Eighteen morbidly obese females undergoing sleeve gastrectomy were followed pre-and post-surgery. Stool from four patients, showing resolved MASLD post-surgery with sustained weight loss, was transplanted into antibiotic treated mice. Mice received pre-or post-surgery stool and were fed a standard or high-fat diet. Bodyweight, food intake, and physiological parameters were tracked weekly. Metabolic parameters were measured post-study termination.

**Results:**

The human study revealed that bariatric surgery led to significant weight loss (*p* > 0.05), decreased inflammatory markers, and improved glucose levels six months post-surgery. Patients with weight loss of 20% or more showed distinct changes in blood metabolites and gut microbiome composition, notably an increase in *Bacteroides*. The mouse model confirmed surgery-induced microbiome changes to be a major factor in the reduction of markers and attenuation of MASLD progression. Mice receiving post-surgery fecal transplants had significantly less weight gain and liver steatosis compared to pre-surgery recipients. There was also a significant decrease in inflammatory cytokines interferon gamma, interleukin 2, interleukin 15, and mig. This was accompanied by alterations in liver immunophenotype, including an increase in natural killer T cells and reduction of Kupfer cells in the post-surgery transplant group.

**Discussion:**

Our findings suggest surgery induced microbial changes significantly reduce inflammatory markers and fatty liver progression. The results indicate a potential causal link between the microbiome and the host immune system, possibly mediated through modulation of liver NKT and Kupffer cells.

## 1 Introduction

The prevalence of obesity in adults has doubled in the last decade alone, confirming that the obesity epidemic of the late 1900s is only worsening (Park et al., 2005; Hruby and Hu, 2015). Obesity is understood as the underlying mechanism for several comorbidities, one being metabolic associated steatotic liver disease (MASLD). MASLD is the leading cause of chronic liver disease and for liver-related mortalities globally (Younossi et al., 2016; Ding et al., 2023). The rates of MASLD in the population have increased in parallel with the rise in obesity, affecting over 25% of the global population (The GBD 2015 Obesity Collaborators, 2017; Pouwels et al., 2022). While the disease itself can be relatively asymptomatic and in some cases, reversible, nearly 25% MASLD cases progress into stages in which the liver becomes overly fibrotic and eventually scarred (Ismail and Pinzani, 2009; Fallowfield and Hayes, 2011; Bashiardes et al., 2016). Given its rise and the detrimental outcomes of MASLD, understanding the underlying mechanisms of MASLD progression, and identifying potential therapeutic strategies for MASLD remains a leading area of research.

Bariatric surgery has been a very well established treatment for eligible patients with both obesity and MASLD. Many studies have shown the beneficial effects of bariatric surgery in MASLD (Gulinac et al., 2023). It is thought the main effect of bariatric surgery on MASLD is through weight loss. However, MASLD improvement can often occur before significant weight loss is achieved (Seeberg et al., 2022). This suggests that the effects of bariatric surgery on MASLD are potentially multifactorial.

The gut microbiome has recently emerged as a critical factor contributing to the progression of MASLD, indicating it to be a promising avenue for investigation (Durack and Lynch, 2019; Afzaal et al., 2022). Dysregulation of the gut microbiota has been associated with compromised gut barrier function, which can contribute to translocation of bacteria and endorse hepatic inflammation, and with the modulation of certain metabolites (Bashiardes et al., 2016; Chopyk and Grakoui, 2020; Afzaal et al., 2022). The gut microbiome also plays an important role in the host innate immune system (Zheng et al., 2020). This is important because several immune cell populations and inflammatory mediators have been implicated in the development and progression of MASLD (Carter and Friedman, 2022; Huby and Gautier, 2022). The liver, as a central organ in the immune response, is constantly exposed to gut-derived antigens and microbial products through the portal circulation, making it susceptible to immune-mediated damage. Studies have shown that various immune cells, including macrophages, T cells, and dendritic cells, infiltrate the liver and contribute to the inflammatory milieu that characterizes MASLD (Ma et al., 2018; Bai et al., 2023).

One specific subset of immune cells, known as natural killer T (NKT) cells, has garnered increasing attention due to their involvement in metabolic conditions like MASLD (Kumar and Delovitch, 2014; Krijgsman et al., 2018). NKT cells are a subset of T cells that express both a T cell receptor and a natural killer cell receptor (Kumar and Delovitch, 2014; Krijgsman et al., 2018; Nilsson et al., 2020). With two receptors, the cells can recognize lipid antigens presented by CD1d molecules, making them critical in lipid metabolism and immune regulation (Dowds et al., 2014). Several studies have shown that NKT cells modulate hepatic lipid metabolism and introduce a pro-inflammatory environment (Nilsson et al., 2020; Zheng et al., 2022). More specifically, the activation of NKT cells has been associated with a prevalence of pro-inflammatory cytokines like tumor necrosis factor-alpha (TNF-α) or interferon-gamma (IFN-γ). Some studies, however, have shown the opposite. Certain subsets of NKT cells have been found to play a protective role, producing anti-inflammatory cytokines like interleukin-4 (IL-4) and interleukin-10 (IL-10) instead (Nilsson et al., 2020).

Given the contradictory results across studies, the precise role of NKT cells in the pathogenesis of MASLD remains an active area of investigation. Understanding the interplay between gut microbiota changes in bariatric surgery and the protective or pathogenic proliferation of NKT cells in the context of liver inflammation could provide novel insight in the underlying mechanisms of MASLD. Therefore, the aim of this study is to investigate the role of the microbiome post-bariatric surgery in MASLD and its effects on NKT cells in the liver disease setting.

## 2 Methods

### 2.1 Human cohort recruitment

A human study using a small cohort design was employed to investigate a causal relationship between the gut microbiome and MASLD after bariatric surgery. Adult patients were recruited from the Bariatric Surgery Program at the University of California, Los Angeles. Patients eligible for the study had to meet the following requirements: biologically female, an intention to undergo sleeve gastrectomy, diagnosis of MASLD, and in line with the criteria listed in the Guidelines for Clinical Application of Laparoscopic Bariatric Surgery of American Gastrointestinal and Endoscopic Surgeons (SAGES Guidelines Committee, 2008). Recruiting patients of the same gender, surgical procedure intent, and from the same program minimized confounding factors that could impact the gut microbiome composition. Diagnosis of MASLD was based on a patient history of non-excessive alcohol use, absence of viral hepatitis, and either pathology and imaging consistent with steatosis or elevated transaminases. Exclusion criteria included cirrhosis, use of medications interfering with or affecting intestinal motility, excessive substance abuse, a history of major gastrointestinal surgeries or procedures, irritable bowel syndrome, inflammatory bowel disease, pregnancy, or recent antibiotic or probiotic use. The demographic information of each participant was collected as well as stool and blood samples before surgery and six months after surgery.

Ethical approval of the study was obtained from the University of California, Los Angeles Institutional Review Board (IRB #13-001552). All patient research procedures followed the principles of the Declaration of Helsinki.

### 2.2 Human cytokine profiling

Fasting blood samples were obtained from patients both before and 6 months after undergoing bariatric surgery. Subsequently, the levels of C-reactive protein (CRP) (Abcam, ab260058) and lipopolysaccharide-binding protein (LBP) (Abcam, ab279407) were quantified using an enzyme-linked immunosorbent assay (ELISA) kit, following the manufacturer’s recommended protocols. The following cytokines were measured: HGF, IL-1beta, IL-6, IL-8, MCP-1, NGF, and TNF-α. Fast blood serum was also analyzed for metabolites using 16S sequencing.

### 2.3 Human serum metabolite profiling

Fasting serum was collected from patient and sent to Metabolon Inc. for processing.

### 2.4 Human stool collection and 16S sequencing

Stool samples were collected from the participants within one week prior to their scheduled surgery, as well as at 6 and 12 months post-surgery. Fresh stool samples were promptly collected and immediately frozen, ensuring preservation at a temperature of-80°C. Subsequently, the frozen stool samples were divided into aliquots for subsequent analysis. DNA extraction was performed using the ZymoBIOMICS DNA Microprep Kit (Zymo Research, United States), following the manufacturer’s instructions.

To target the V4 region of the 16S ribosomal RNA gene, PCR amplification was conducted utilizing the 515\u00B0F-806 R primer set (Tong et al., 2014). Following the amplification step, the samples underwent 250 × 2 paired-end sequencing using an Illumina HiSeq platform (Illumina, San Diego, CA, United States) (Zheng et al., 2022). The resulting raw fastq files were subjected to processing using the DADA2 pipeline in R, employing the SILVA 132 database and default parameters for taxonomy assignment (Callahan et al., 2016).

After initial pre-processing in R using DADA2, the data were subsequently integrated into QIIME 2 version 2019.10 (Bolyen et al., 2019). To ensure data quality, amplicon sequence variants (ASVs) that were sparsely represented were filtered out if they were not present in at least 15% of all samples, consistent with our previous publications (Dong et al., 2020; Hussain et al., 2020). The sequence depths ranged from 60,710 to 269,258 per sample.

### 2.5 Statistical analysis of human data

Physiological metrics were analyzed after calculating the mean values and standard deviations of quantitative demographic information, including age, body weight, and body mass index (BMI). The Shapiro–Wilk test was done to test for normality of continuous data from the human trial. Data deemed to be not normally distributed if the Shapiro–Wilk test had a *p*-value <0.05. Parametric test were done on normally distributed data and non-parametric tests were done on non-normally distributed data. A paired t-test was used to statistically compare respective mean values of normally distributed data. A paired Wilcoxon signed-rank test was used to statistically compare non-normally distributed data: body weight, BMI, fasting glucose, and inflammatory cytokines in patients before and after surgery. A Fisher’s exact test was used to assess categorical demographic information, like ethnicity and race.

Changes to microbial composition were analyzed using several metrics. Alpha diversity was computed using the Shannon Index within the QIIME2 framework (Bolyen et al., 2019). The Shannon index incorporates both species and evenness. The microbiome dataset was rarefied to a standardized sequence depth of 60,710 reads. The statistical significance of the Shannon Index was determined using analysis of variance (ANOVA) after confirming normality using the Shapiro–Wilk test, adjusting for subject ID as a covariate. Beta diversity was analyzed using the robust Aitchison Distance Metric within the DEICODE package of QIIME2 (Martino et al., 2019). The Aitchison Distance Metric has demonstrated superior discriminatory capacity compared to other metrics, such as UniFrac or Bray-Curtis (Lozupone and Knight, 2005; Martino et al., 2019). Beta diversity was evaluated using permutational multivariate analysis of variance, specifically employing the ‘adonis’ package in R (Version 4.1.2), adjusting for subject ID as a covariate (Dong et al., 2021). Differential abundance analysis of genera was computed using MaAsLin2, in which subject ID was a random effect (Mallick et al., 2021).

Metabolite data was transformed using a median-scale normalization approach–one in which each metabolite value was divided by its median value. Metabolite levels over different time points were statistically compared using ANOVA, adjusting for subject ID as a covariate. *p*-values were adjusted for multiple hypothesis testing using the Benjamin-Hochberg correction method (Benjamini and Hochberg, 1995; Glueck et al., 2008). A heatmap was generated using the ‘ggplots’ and heatmap.2 function in R to visually depict significant metabolites. *p*-values deemed significant were those less than 0.05, adjusted for multiple hypothesis testing if appropriate.

### 2.6 Mouse model

A mouse model was designed to engraft the microbiome of human patients into antibiotic treated mice. 64 C57/B16 male mice between 6–8 weeks old at the start of the study were separated into 16 cages with four mice in each cage. Since we were evaluating the role of the immune system in our model, ferm-free mice were not used because of their altered immune systems (Kennedy et al., 2018). Eight cages received donor stool collected prior to surgery and eight cages received donor stool collected six months post-surgery. Within those time point groups, half was assigned a standard double-irradiated diet (10% fat by kcal, 0% fructose, and 0% cholesterol) and the other was assigned a Western high-fat double-irradiated diet (40% fat, 20% fructose, 2% cholesterol) for the duration of the study. Each cage was provided with individually packaged 1-kg bags of food, with a new bag introduced during each cage change.

A combination of systemically absorbed antibiotics (ampicillin, cefoperazone, and clindamycin) and non-systemically absorbed antibiotics (ertapenem, neomycin, and vancomycin) were given on alternating weeks in their drinking water for 21 days prior to the beginning of the study. This process has been shown to lead to stable engraftment of human microbiota in non-germ-free animals. The concentration of antibiotics in the mice’s water was maintained at 1 g/L *ad libitum*.

Stool samples for fecal transplant were prepared using donor stool from four patients who met the following criteria: diagnoses of MASLD prior to surgery that resolved after surgery and achieved significant and sustained weight loss of at least 20% six months following surgery. Resolution of MASLD was determined either by biopsy or by imaging. Approximately 1 g of frozen stool per time point and donor was resuspended in 15 mL of pre-reduced PBS and filtered through a 100 μm filter. Mice were then orally administered 200 μL of this solution. The microbial concentration of the preparation was determined using a Petroff-Hausser counting chamber, and approximately 10^10 cells were administered during each gavage procedure.

This research was conducted in compliance with the guidelines and approval of the UCLA Animal Research Committee and the Institutional Animal Care and Use Committee.

### 2.7 Mouse body composition and glucose tolerance testing

The body weight of each mouse and average food intake per mouse were recorded once a week. At the end of the 90-day period, a body composition analysis, including lean body mass and fat mass, was conducted using an EchoMRI machine. To assess glucose tolerance, mice were fasted for 9 h and then injected intraperitoneally 2 g/kg of glucose. Serum glucose levels were measured at 0, 30 min, 60 min, and 90 min using a glucometer.

### 2.8 Mouse sample processing

Mice were fasted 9 h before being euthanized. Isoflurane was used as the primary method for euthanasia. Animals were exposed to a 10% concentration of isoflurane. Heart puncture and phlebotomy were carried out as a secondary method. Serum samples were obtained via heart puncture, and the liver and gastrointestinal tract were collected.

### 2.9 Liver steatosis staining and quantification

Frozen OCT-embedded liver tissue was stained with Oil red O. The quantification of staining was performed using ImageJ. Hematoxylin and eosin staining was conducted by the histology core at the University of California, Los Angeles. A blinded pathologist calculated MASLD activity scores based on the hematoxylin and eosin staining. Liver triglyceride content was quantified using a calorimetric triglyceride assay kit (Abcam ab65336), per the manufacturer protocol.

### 2.10 Serum inflammatory markers and hepatic immune flow cytometry

IL-6, TNF-α, CCL2, IL-4, and TGF-β were quantified using the Millipore Milliplex Cytokine Kit (Melamud et al., 2022). This kit also includes other cytokines such as IL-1β, IL-2, IL-15, IL-10, IL-12p70, IL-13, IL-17A, IFN-γ, MCP-1, MIP-1a, MIP-1b, GM-CSF, CCL3, CCL4, MIG, and CCL5, which play critical roles in various inflammatory and immune responses, providing valuable insights into MASLD and HCC development (Kennedy et al., 2018).

The activation of hepatic immune cells was assessed using flow cytometry and cell surface marker staining (Hildreth et al., 2023). The liver of each mouse was perfused with PBS and EDTA, followed by incubation with HBSS containing collagenase IV and DNAase I. Each liver was homogenized, filtered through a cell strainer, and centrifuged. The resulting pellet was resuspended and separated using a Percoll gradient. Flow cytometry antibodies targeting specific markers were used, including Alexa Fluor® 488 anti-mouse Ly-6C, Alexa Fluor® 647 anti-mouse F4/80, APC/Cyanine7 anti-mouse CD45.2, Brilliant Violet 650™ anti-mouse CD3, Brilliant Violet 750™ anti-mouse/human CD11b, PE anti-mouse α-GalCer:CD1d complex, PE/Dazzle™ 594 anti-mouse CD335 (NKp46), PerCP/Cyanine5.5 anti-mouse CD4, Brilliant Violet 421™ anti-mouse/human CD45R/B220, and Brilliant Violet 510™ anti-mouse CD8a. The antibodies were obtained from BioLegend.

The flow cytometry panel allowed for the characterization of various immune cell populations, including T cells (CD3, CD4, and CD8a), B cells (CD45R/B220), macrophages (F4/80), monocytes (Ly-6C), and NKT cells.

### 2.11 Statistical analysis for mouse data

A paired *t*-test was used to statistically compare groups. Shapiro–Wilk test was done to test for normality.

## 3 Results

### 3.1 Bariatric surgery leads to decreased obesity markers

**Figure 1:**
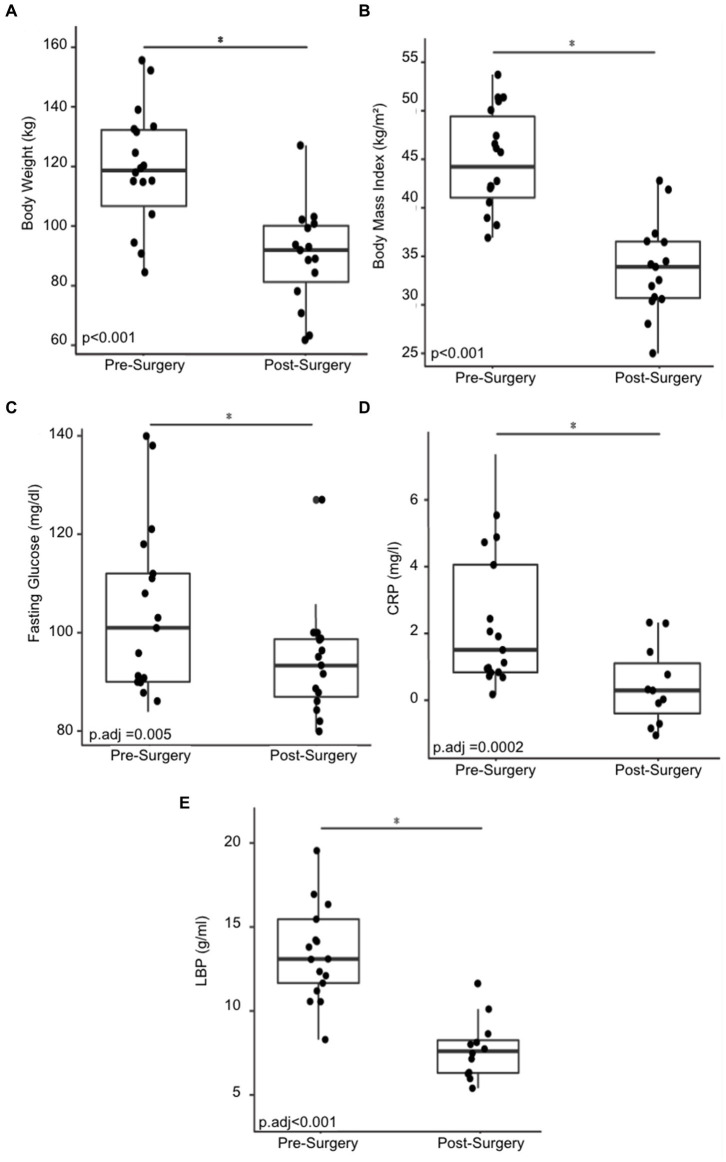
Changes in physical and physiological metrics post-bariatric surgery. **(A)** Body weight of 18 cohort members before and six months post-surgery. **(B)** BMI before and six months post-surgery. **(C)** Fasting glucose before and six months post-surgery. **(D)** C-reactive protein (CRP) before and six months post-surgery. **(E)** Change in lipopolysaccharide binding protein (LBP) before and six months post-surgery. “*” indicates *p*-value<0.05.

Eighteen morbidly obese females were recruited for the human cohort study. Their demographic and physical characteristics were recorded (Table 1). Non-Hispanic White individuals made up 44.4% of the cohort and Hispanic individuals made up 38.9%. Asians made up 11.1% of the cohort and African Americans made up 5.6%. The mean pre-surgery BMI was 44.7 ± 4.9 and the mean pre-surgery body weight 118.5 ± 18.8 kg. The cohort exhibited a statistically significant reduction in BMI and body weight six months after bariatric with a mean post-surgery bodyweight of 89.7 ± 16.9 kg (Figure 1A), and a post-surgery BMI of 33.9 ± 4.8 (*p* < 0.001) (Figure 1B). Blood fasting glucose decreased by 7.9% and the inflammatory markers C-reactive protein (CRP) and lipopolysaccharide binding protein (LBP) decreased by approximately 87.5 and 42.3%, respectively, six months post-surgery (Figures 1C–E).

**Table 1:** Cohort characteristics.

Race and ethnicity (%)	
Non-Hispanic White	44.4
Hispanic	38.9
Asian	11.1
African American	5.6

Average (SD) (*n* = 18)	Six months pre-surgery	Six months post-surgery	*p*-value
Age (yr)	37.1 (9.4)	37.1 (9.4)	–
Body Mass Index	44.7 (4.9)	33.9 (4.8)	<0.001
Weight (kg)	118.5 (18.8)	89.7 (16.9)	<0.001

The demographic and physical characteristics of 18 females recruited for the human cohort study. SD: Standard Deviation.

### 3.2 Bariatric surgery reduces blood metabolites and introduces changes in gut microbiome composition

**Figure 2:**
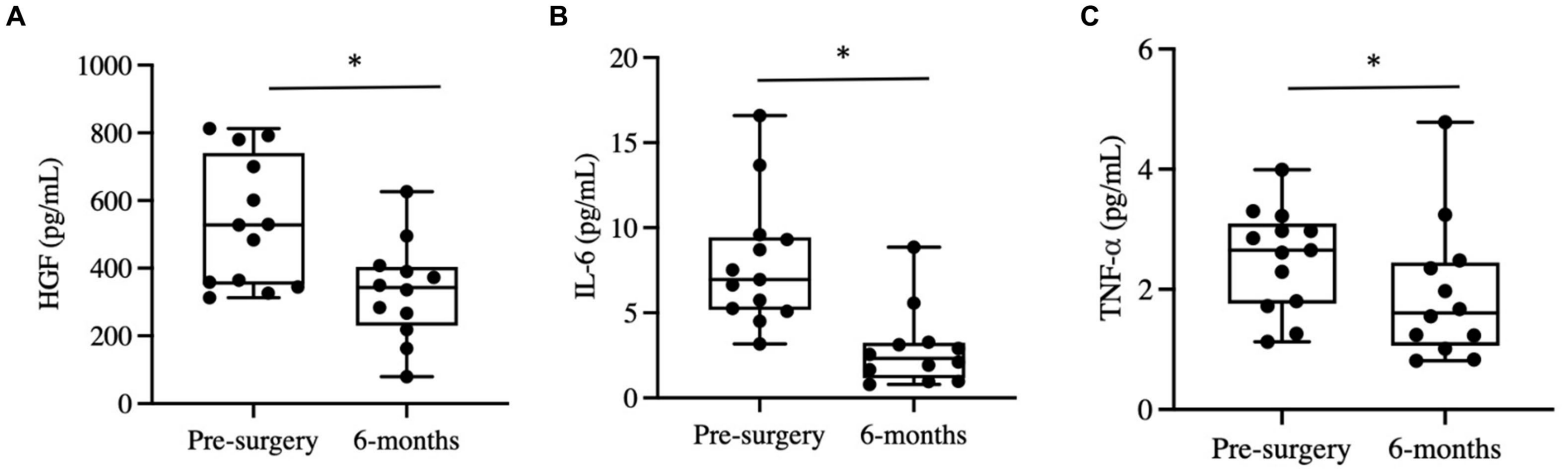
Changes in blood cytokines post-bariatric surgery. **(A)** Hepatocyte growth factor (HGF) before and six months post-surgery. **(B)** Interleukin-6 before and six months post-surgery. **(C)** Tumor necrosis factor alpha (TNF-α). “*” indicates *p*-value<0.05.

Six months post-bariatric surgery, patients demonstrated a noteworthy decline in several blood metabolites. Specifically, hepatocyte growth factor (HGF), interleukin-6 (IL-6), and tumor necrosis factor alpha (TNF-α) levels significantly decreased (Figures 2A–C). No significant differences were observed in interleukin-1 beta (IL-1B), interleukin-8 (IL-8), monocyte chemoattractant protein 1 (MCP1), or nerve growth factor (NGF) concentrations.

**Figure 3:**
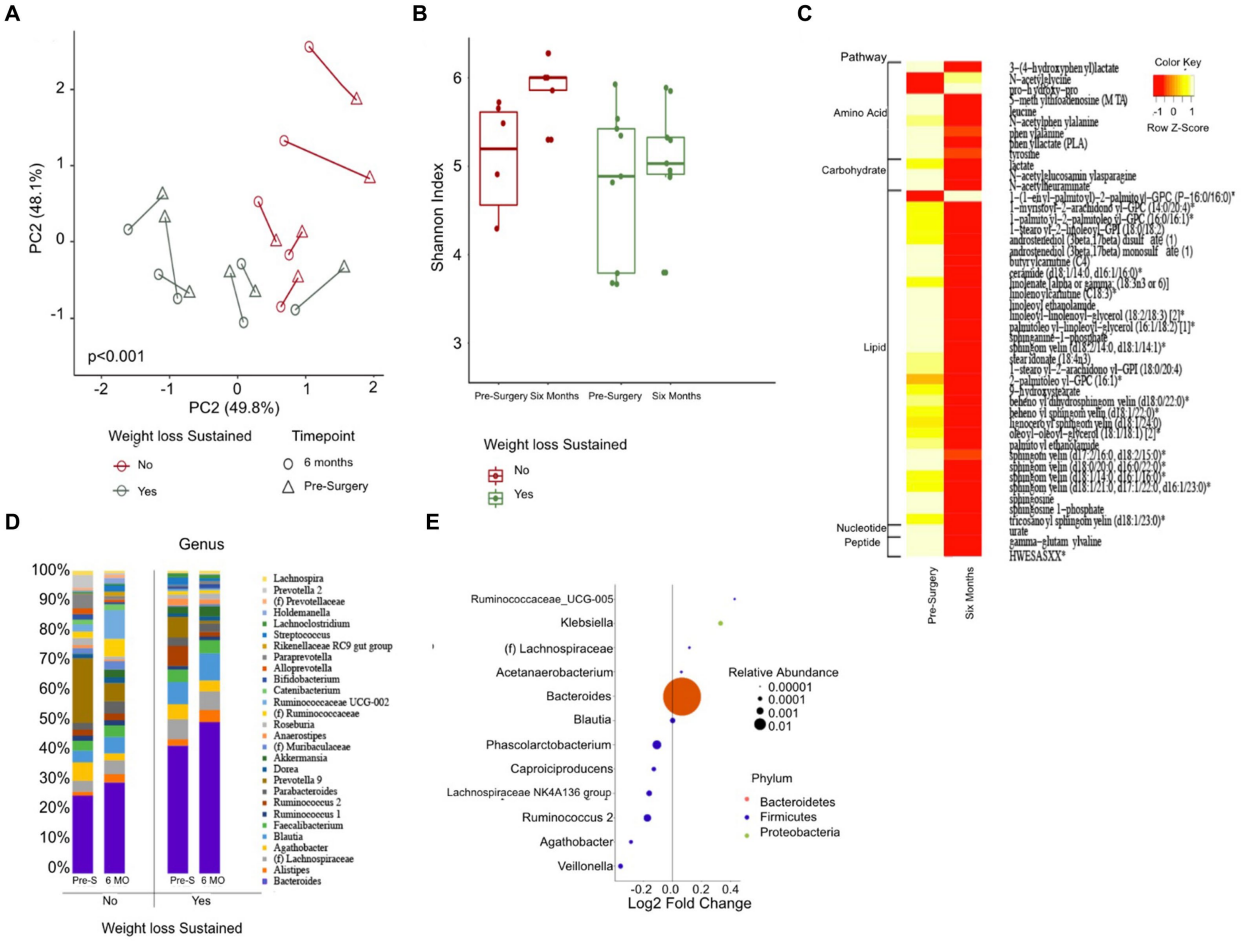
Changes in gut microbiome composition and serum metabolites following bariatric surgery. **(A)** Principal coordinate analysis plot (PCoA) measuring beta diversity. Each patient sample is indicated by either a triangle for pre-surgery or circle for six-months post-surgery, color-coded in either green for sustained weight loss or red for otherwise. Shapes are connected by a line to indicate an individual patient’s sample over time. **(B)** Shannon Index quantifying alpha diversity. **(C)** Heatmap of serum metabolites before and six months post-surgery, categorized by major biochemical pathway. Only metabolites that were significantly different are shown. **(D)** Stacked bar plot showing the average relative abundance of each genera. Only genera with a relative abundance of at least 1% are included. **(E)** Results of differential abundance testing by MaAsLin, showing genera that are different from baseline. The test is adjusted for sustained weight loss (SWL) with patient ID as a random effect.

Upon analysis of beta diversity, patients who maintained a sustained weight loss of 20% or greater from their baseline had distinct differences in their gut microbiomes both at baseline and six months post-surgery compared to those who did not sustain weight loss (Figure 3A). Patients that sustained weight loss also exhibited significantly lower alpha diversity both at baseline and post-surgery (Figure 3B). There was no significant difference between alpha or beta diversity between individual patients’ samples pre-surgery of six months afterwards. Patients who sustained weight loss had higher levels of *Bacteroides* at baseline, with an upward trend in relative abundance over the duration of the study while patients who did not sustain weight loss over time had lower levels of *Bacteroides* at baseline and a relatively unchanged level overtime.

46 blood metabolites were identified as significantly different at the six-month post-surgery time point when compared to their baseline values. Of these, 31 metabolites were found to be linked to lipid metabolism, while 9 were associated with amino acid metabolism (Figure 3C).

Gut Differential Analysis reveals 12 patients who sustained weight loss to have altered genera six months post-surgery. *Bacteroides*, which had the largest relative abundance amongst both groups, increased noticeably after the sleeve gastrectomy procedure; 22.5% at baseline to 25.5% for patients without sustained weight loss and 36.6 to 42.8% six months after surgery for patients who did (Figures 3D,E). Other genera that were altered pre-and post-surgery include *Acetanaerobacterium, Phascolarctobacterium, Ruminococcaceae UCG-005, Ruminococcus 2, Klebsiella, Blautia, Lachnospiraceae NK4A136 group, Lachnospiraceae NK4A136 group, (f) Lachnospiraceae, Capriociproducens, Agathobacter, and Veillonella*.

### 3.3 Bariatric surgery-induced gut microbiome changes in a preventative mouse model result in body weight reduction and attenuation of MASLD progression

**Figure 4:**
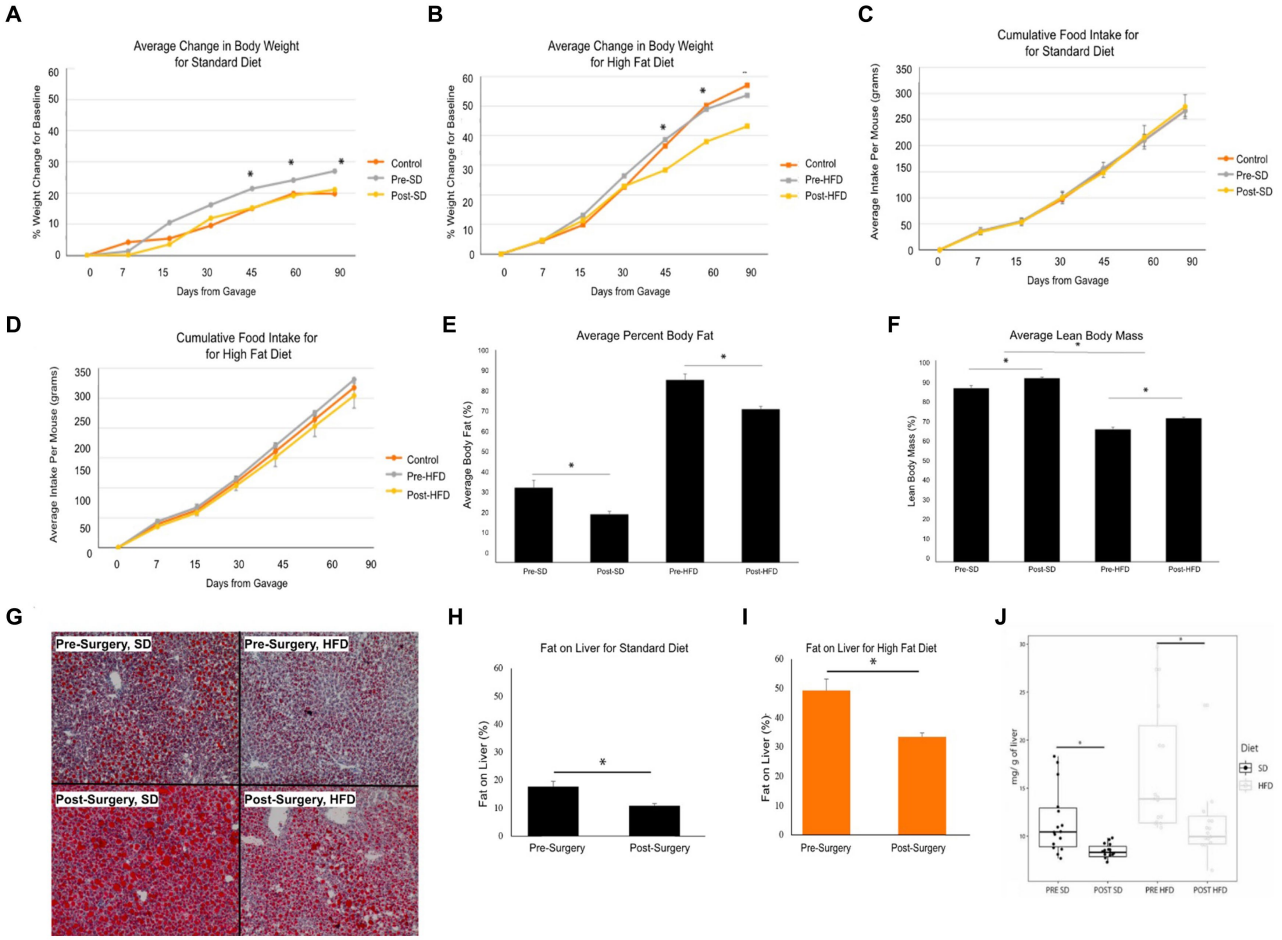
Bariatric surgery induced microbiome changes protect against MASLD progression in mice. **(A)** Average change in body weight for mice on a standard diet (SD) over 90 days. Orange indicates the control group with a vehicle gavage; gray indicates the group which received the pre-surgery stool transplant; yellow indicates the group that received the post-surgery stool transplant. **(B)** Average change in body weight for mice on high fat diet (HFD) over 90 days. **(C)** Cumulative food intake for mice on a standard diet over 90 days. **(D)** Cumulative food intake for mice on a high fat diet over 90 days. Comparison of average percent body fat between mice receiving pre-surgery versus post-surgery stool transplant on either diet following 90 days of gavage. **(E)** Comparison of average percent body fat of mice receiving pre-surgery versus post-surgery stool transplant on either diet following 90 days of gavage. **(F)** Comparison of average lean body mass of mice receiving pre-surgery versus post-surgery stool transplant on either diet following 90 days of gavage. **(G)** Images of Oil red o-stained liver samples from pre-surgery or post-surgery transplants on either diet. **(H)** Quantification of fat on liver for mice on standard diet using ImageJ. **(I)** Quantification of fat on liver from mice on high fat diet using ImageJ. **(J)** Quantification of mouse liver triglycerides. “*” indicates *p*-value<0.05.

Stool samples were collected from four donors who met the criteria for fecal transplantation. (Table 2). Mice with baseline donor stools (donor stool collected pre-surgery) gained significantly more weight on either diet compared to those who received the donor stool six months post-surgery (Figures 4A,B). No significant difference was seen in the average food intake in either of the two groups (Figures 4C,D). A measurement of body fat percentage using EchoMRI showed that mice on either diet who received the post-surgery stool had significantly lower body fat percentage and higher average lean body mass compared to their respective pre-surgery groups (Figures 4D,E). Histological analysis of the liver revealed that pre-surgery groups on either diet had significantly elevated hepatic steatosis compared to their respective post-surgery groups (Figure 4G). This was verified both by quantification of oil red o lipid staining using ImageJ and quantification of liver triglycerides (Figures 4H–J).

**Table 2:** Microbiome donor characteristics.

Donor	Race/ethnicity	Age	Body mass index		% Body weight loss at six months	Fatty liver resolution on imaging
			Pre-surgery	Post-surgery		
A	Non-Hispanic White	25	51.0	37.1	20.1	Yes
B	Hispanic	21	42.2	30.9	23.3	Yes
C	Asian	45	36.9	25.1	35.3	Yes
D	Hispanic	43	38.2	31.0	20.2	Yes

The demographic and physical characteristics of four cohort members who had fatty liver disease prior to surgery that resolved after six months after treatment.

**Figure 5:**
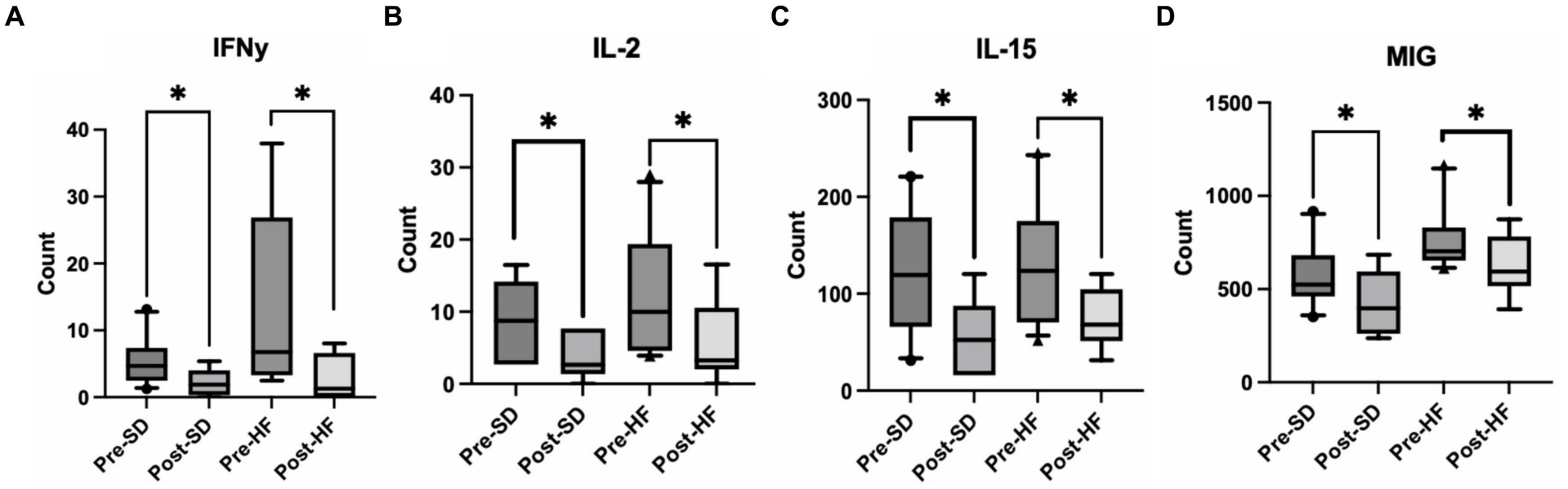
Post bariatric microbiome changes alters inflammatory cytokines in the serum. These four groups are consistent across graphs: Mice who received pre-surgery stool and were on a standard diet (Pre-SD); mice who received post-surgery stool and were on a high fat diet (Pre-HFD); mice who received post-surgery stool and were on a standard diet (Post-SD); mice who received post-surgery stool and were on a high fat diet (Post-SD). Comparison of **(A)** interferon gamma (IFN-y), **(B)** interleukin-2 (IL-2), **(C)** interleukin-15 (IL-15), **(D)** Mig between the pre-and post-surgery groups within their respective diets. “*” indicates *p*-value<0.05.

Mice who received the post-surgery stool had several significantly lower inflammatory cytokines in their serums as compared to mice who received the pre-surgery stool, regardless of diet. Specifically, interferon gamma (IFN-y), interleukin-2 (IL-2), interleukin 15 (IL-15), and mig levels were significantly lower (Figures 5A–D). No other cytokines tested were significantly different.

### 3.4 Bariatric surgery-induced gut microbiome alters immunophenotype and reduces inflammation

**Figure 6:**
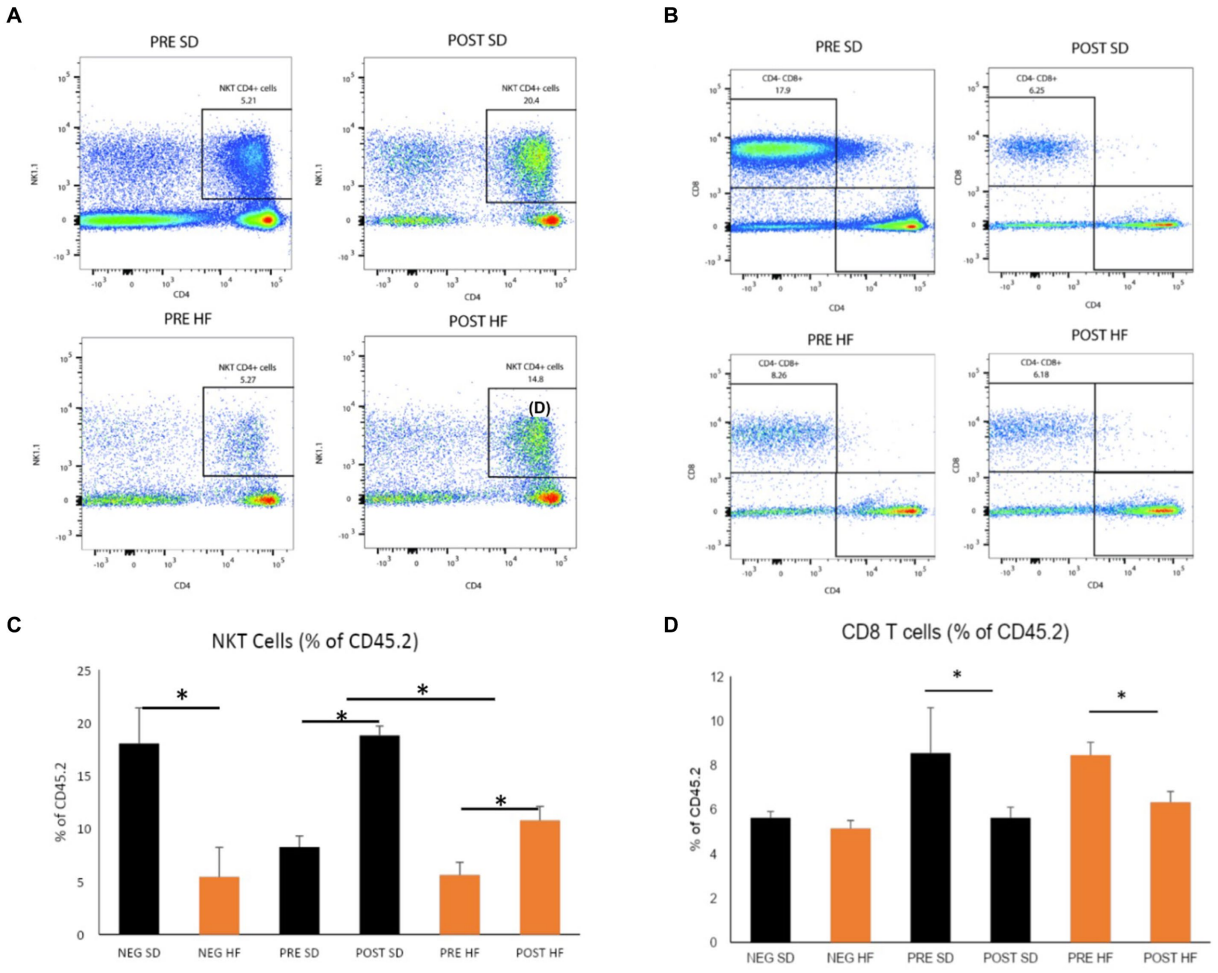
Natural killer T and CD8 T cells are altered by microbial changes post-bariatric surgery. Flow cytometry data displayed in density plot for four groups: Mice who received pre-surgery stool and were on a standard diet (Pre SD); mice who received post-surgery stool and were on a high fat diet (Pre HFD); mice who received post-surgery stool and were on a standard diet (Post SD); mice who received post-surgery stool and were on a high fat diet (Post SD). **(A)** Comparison of NKT cell count in black square box labeled “NKT CD4+ Cells.” Mice who received post-surgery stool had a higher count of NKT cells compared to the pre-surgery group on either diet. **(B)** Comparison of CD8 T cells cell count in black square box labeled “CD4+ CD8 + .” Mice who received post-surgery stool had a lower count of CD8 T cells compared to the pre-surgery group on either diet. **(C)** Quantification by percentage of density plot **(A)**. **(D)** Quantification by percentage of 606 density plot **(B)**. “*” indicates *p*-value<0.05.

**Figure 7:**
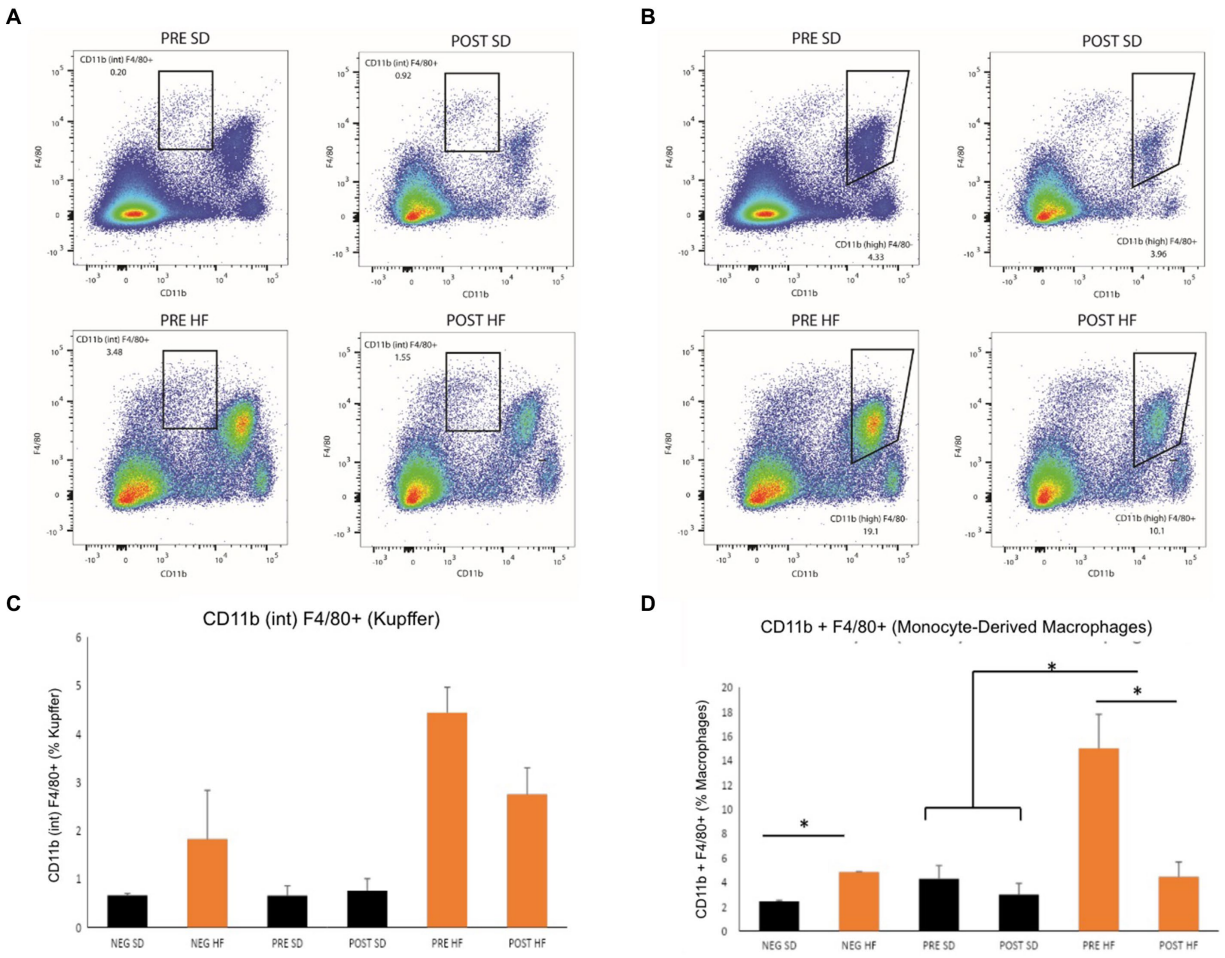
Mice in pre-surgery group have higher level Kupffer cells and macrophages. Flow cytometry data displayed in density plot for four groups: Mice who received pre-surgery stool and were on a standard diet (Pre SD); mice who received post-surgery stool and were on a high fat diet (Pre HFD); mice who received post-surgery stool and were on a standard diet (Post SD); mice who received post-surgery stool and were on a high fat diet (Post SD). **(A)** Comparison of Kupffer cell count in black square box labeled “CD11b (int) F4/80.” Mice who received post-surgery stool had a visually lower quantity of Kupffer cells than mice in the pre-surgery group on either diet. **(B)** Comparison of Monocyte-Derived macrophage count in black square box labeled “CD11b (high).” Mice who received post-surgery stool had a lower count of macrophages compared to the pre-surgery group on either diet. **(C)** Quantification by percentage of density plot **(A)**. **(D)** Quantification by percentage of 606 density plot **(B)**. “*” indicates *p*-value<0.05.

The immunophenotype of the livers reflected an increase in natural killer T cells (NKT cells) in both the post-surgery groups compared to their respective pre-surgery groups, but the groups receiving a high fat diet demonstrated a significant decrease overall in the percentage of NKT cells (Figures 6A,C). Cytotoxic T-cells (CTLs) were lower in the post-surgery group on both diets with no significant differences in the quantity of CTLs between diet groups (Figures 6B,D). A decrease in Kupffer cells was evident in the post-surgery groups on either diet with a notable elevation of Kupffer cell quantity overall in the groups receiving the high fat diet (Figures 7A,C). There was a significant decrease in inflammatory macrophages of the post-surgery group on either diet, and a notable increase in inflammatory macrophages overall in the groups receiving the high fat diet (Figures 7B,D).

## 4 Discussion

Bariatric surgery is one of the leading treatment options for a long-term positive outcome for obese individuals with MASLD. The primary purpose of this study was to demonstrate that the microbiome is partially responsible for MASLD improvement following bariatric surgery. The long-term positive trends observed in the human cohort reflected those seen in previously published data on more extensive cohorts and confirm that the treatment is effective (Courcoulas et al., 2014). Notably, individuals who underwent sleeve gastrectomy achieved a total body weight loss of 22.7% and a decrease in BMI of 21.6% six months after surgery. This decrease was associated with significant downwards trends in concentrations of C-reactive protein and lipopolysaccharide-binding protein, both notable inflammatory markers associated with obesity. Previous studies examining visceral adipocyte tissue in obese patients have revealed that expressions of hepatocyte growth factor (HGF), interleukin-6 (IL-6), and tumor necrosis α (TNF-α) are significantly elevated in obese patients compared to lean ones (Park et al., 2005; Gletsu-Miller and Wright, 2013; Muratsu et al., 2017). Likewise, studies incorporating bariatric surgery and its role in obesity show a decrease in adiposity and in serum levels of IL-6, and TNF-α (Casimiro et al., 2020). Elevated levels of HGF have also been found to be associated with increased insulin resistance, a marker of severe obesity (Muratsu et al., 2017). As anticipated with the differences in physiological markers for patients in our cohort, measurements of inflammatory cytokine markers HGF, IL-6, and TNF-α were significantly lower six months after surgery compared to before. These results match the trends of more extensive bariatric surgery trials referenced.

A comparison of the microbiome composition of individuals before and after surgery reveals significant long-term differences. *Bacteroides* have previously been shown to have a negative correlation with physiological markers of obesity–body weight, BMI, and body fat (Aoun et al., 2020; Zsálig et al., 2023). The findings in this study concur with these trends; individuals in our study had increased levels of *Bacteroides* after surgery. Those who sustained weight loss after surgery had a more pronounced increase compared to those who did not. The exact mechanisms by which *Bacteroides* are correlated with weight loss remain unclear. However, multiple studies propose that these species may contribute to weight management through their potential involvement in gut-barrier integrity and metabolism (Ghosh et al., 2021; Zafar and Saier, 2021). *Bacteroides* have previously been shown to promote tight junction expression between epithelial cells in the small intestine, reducing permeability and potentially hindering the translocation of pro-inflammatory pathogens from the gut lumen into the bloodstream (Zhou et al., 2022). Consequently, an increase in the population could protect against systemic inflammation, a hallmark of obesity-related dysfunction. *Bacteroides* are also known for their ability to synthesize SCFAs as byproducts of complex dietary fibers. SCFAs are positively associated with glucose homeostasis and satiety (Ghosh et al., 2021; Zafar and Saier, 2021). Ghosh et al. (2021) particularly emphasized the potential impact of *Bacteroides-*synthesized SCFAs on insulin sensitivity. Several mouse studies and human studies have shown that SCFA are altered after bariatric surgery, with butyrate and propionate being positively associated with weight loss.

In order to demonstrate a causal relationship between changes in the gut microbiome induced by bariatric surgery and the progression of MASLD, fecal samples pre-surgery and post-surgery of cohort members who had resolution of MASLD post-surgery were transplanted into antibiotic treated mice. Mice who had received the pre-surgery microbiome showed increased body weight and a more rapid increase in body fat complemented with more severe progressed MASLD and fat on the liver, demonstrated in the histological staining and quantification. These trends were observed regardless of diet with no significant differences in food intake amongst those receiving the pre-surgery or post-surgery transplant, matching those of the findings in *Tremaroli et al.*; mice receiving the pre-surgery transplant had gained greater weight than those receiving the post-surgery transplant with no difference in respiratory levels, activity, or average food intake (Tremaroli et al., 2015). Mice receiving the post-surgery transplant also had a significant decrease in interferon gamma (IFN-y), interleukin-2 (IL-2), interleukin 15 (IL-15), and mig levels in their serum as compared to the pre-surgery transplant group. The active role of these cytokines is linked to growth of hepatocytes and a more rapid progression of MASLD (Chang et al., 2015; Duan et al., 2022). Moreover, in *Tarantino et al.*, IL-15 is identified as a predictor for intima-media thickness independent of age, suggesting the role of the cytokine in the onset and progression of atherosclerosis in obese individuals (Tarantino et al., 2021). While the exact mechanism by which the gut microbiome can modulate the development of obesity and MASLD remains unknown, we propose that the microbiome is affecting the progression of MASLD via the host innate immune system, specifically via natural killer T cells (NKT).

Hepatic lipid accumulation triggers a complex interplay of immune responses centered around Kupffer cells, which subsequently contributes to the progression of fibrosis, cirrhosis, and ultimately hepatocyte death (Chiang et al., 2011; Krijgsman et al., 2018). At the forefront of the immune defense, the innate immune system orchestrates rapid reactions through lymphocyte infiltration and localized inflammation. NKT cells, recognized by their expression of both CD3 and a unique natural killer cell marker, play a distinct role in this context. These cells exhibit an unconventional antigen recognition mechanism, responding to lipid antigens presented by CD1d molecules. This specialized characteristic suggests their potential involvement in the pathogenesis of fatty liver diseases, where lipid accumulation serves as a hallmark feature.

In the studies presented in this paper, mice with the pre-surgery microbiome had higher weight gain and lower levels of NKT cells, worsened fatty liver pathogenesis, and increased insulin resistance as compared to their counterparts. This trend is consistent with those demonstrated in earlier findings. Previous murine studies consistently reveal a decrease in NKT cells in obese phenotypes (Lynch et al., 2012; Subramanian et al., 2018). It should be noted, however, that there exists unclear information about the role of NKT cells in obesity; while murine models consistently show a depletion in NKT cells, several human studies have conflicted results (Ohmura et al., 2010). This is possibly due to the stage at which human MASLD is clinically evaluated compared to murine studies. Existing murine models focus on the inhibitory role of NKT cells in early stages of fibrosis while later human studies suggest the promotion of progression in later stages of fibrosis (Teige et al., 2010; Lee et al., 2022). A recently published paper using knockdown and overexpression of CD1d, showed that overexpression of CD1d was protective in human and mouse MASLD models (Lei et al., 2024).

Kupffer cells, the resident macrophages of the liver, are regulated by the recruitment of inflammatory cytokines by cytotoxic T cells (CTL) (Kolios, 2006; Zhang and Bansal, 2020). Kupffer cells were significantly decreased in post-surgery mice in either group, indicating a relationship between the transplanted microbiome and macrophage activation. Active macrophages further enhance the pro-inflammatory environment by amplifying the cytokine response (Kolios, 2006). It is likely that dysbiosis in obesity phenotypes may contribute to the recruitment of cytokines by CTL and subsequent activation of Kupffer cells (Krijgsman et al., 2018; Zhang and Bansal, 2020). This could have a dual impact on the increase in cytokine secretion and depletion in NKT cells.

In conclusion, the findings in this study suggest that bariatric surgery induces microbiome changes that interacts with the host innate immune system, influencing the progression of MASLD. The human cohort study confirms that the microbiome of an obese individual undergoes distinct changes after bariatric surgery both physically and with biological markers of obesity. These findings were replicated in the mouse model using fecal transplants from qualifying cohort members that recovered from MASLD. Mice who received the pre-surgery stool reflected an obese phenotype with greater inflammatory markers than those who received the post-surgery stool, regardless of diet. Quantification of innate immune cells using flow cytometry suggests a depletion of NKT cells in mice receiving the pre-surgery stool, paired with an increase in pro-inflammatory cytokines, findings that were opposite for the post-surgery group. These findings suggest that NKT cells may have a protective role early on against the development MASLD.

### 4.1 Limitations

While our study offers valuable insights into the interplay between microbiome and host innate system on obesity and MASLD, several limitations should be considered. First, despite the observed association between the study phenotypes and their respective immune profile, the exact molecular mechanisms underlying the influence of microbiome changes on NKT cell signaling remain a subject of ongoing investigation. While we tried to explore as many immune cells as we could, not all immune cells were studied. For example, regulatory T cells play a significant role in inflammation and may play a role in MASLD. Therefore, it is possible that other immune cells not measured may play a critical role between the microbiome and MASLD. It should also be noted that microbial transfer has pleiotropic effects that extend beyond the scope of this study. These effects could include, but are not limited to, alterations to bile acids, SCFAs, and hormone signaling pathways. Future studies should involve manipulating NKT cell activity through knockdown and overexpression techniques within a similar microbial transfer model. This will help determine whether NKT cell expression is a crucial mediator between the gut microbiome and MASLD.

## Data availability statement

Raw sequencing data from the human cohort are available on the NIH NCBI BioProject (PRJNA885868) https://www.ncbi.nlm.nih.gov/bioproject/?term=PRJNA885868.

## Ethics statement

The studies involving humans were approved by University of California, Los Angeles Institutional Review Board (IRB #13-001552). The studies were conducted in accordance with the local legislation and institutional requirements. The participants provided their written informed consent to participate in this study. The animal study was approved by UCLA Animal Research Committee and the Institutional Animal Care and Use Committee. The study was conducted in accordance with the local legislation and institutional requirements.

## Author contributions

SS: Visualization, Writing – original draft, Writing – review & editing. WK: Investigation, Visualization, Writing – original draft, Writing – review & editing. JY: Methodology, Investigation, Writing – original draft, Writing – review & editing. CC: Methodology, Investigation, Writing – original draft, Writing – review & editing. NA-J: Investigation, Writing – original draft, Writing – review & editing. VL: Investigation, Writing – original draft, Writing – review & editing. AB: Investigation, Writing – original draft, Writing – review & editing. YC: Investigation, Writing – original draft, Writing – review & editing. ED: Investigation, Writing – original draft, Writing – review & editing. ZL: Methodology, Writing – original draft, Writing – review & editing. EM: Methodology, Funding Acquisition, Writing – original draft, Writing – review & editing. JP: Conceptualization, Writing – original draft, Writing – review & editing. CS: Methodology, Investigation, Supervision, Funding Acquisition, Writing – original draft, Writing – review & editing. SP: Visualization, Writing – original draft, Writing – review & editing. DZ: Visualization, Writing – original draft, Writing – review & editing. ML: Writing – original draft, Writing – review & editing. LH: Visualization, Writing – original draft, Writing – review & editing. JJ: Conceptualization, Visualization, Project Administration, Supervision, Writing – original draft, Writing – review & editing. TD: Conceptualization, Methodology, Investigation, Visualization, Funding Acquisition, Project Administration, Supervision, Writing – original draft, Writing – review & editing.
